# Complete Polar Lipid Profile of Kefir Beverage by Hydrophilic Interaction Liquid Chromatography with HRMS and Tandem Mass Spectrometry

**DOI:** 10.3390/ijms26031120

**Published:** 2025-01-28

**Authors:** Giovanni Ventura, Mariachiara Bianco, Ilario Losito, Tommaso R. I. Cataldi, Cosima D. Calvano

**Affiliations:** 1 Dipartimento di Chimica, Università degli Studi di Bari Aldo Moro, via Orabona 4, 70126 Bari, Italy; giovanni.ventura@uniba.it (G.V.); mariachiara.bianco@uniba.it (M.B.); ilario.losito@uniba.it (I.L.); tommaso.cataldi@uniba.it (T.R.I.C.); 2 Centro Interdipartimentale SMART, Università degli Studi di Bari Aldo Moro, via Orabona 4, 70126 Bari, Italy

**Keywords:** LC-ESI-tandem MS, food, phospholipids, kefir, fatty acyl chains

## Abstract

Kefir, a fermented milk product produced using kefir grains, is a symbiotic consortium of bacteria and yeasts responsible for driving the fermentation process. In this study, an in-depth analysis of kefir’s lipid profile was conducted, with a focus on its phospholipid (PL) content, employing liquid chromatography with high-resolution mass spectrometry (LC-HRMS). Nearly 300 distinct polar lipids were identified through hydrophilic interaction liquid chromatography (HILIC) coupled with electrospray ionization (ESI) and Fourier-transform orbital-trap MS and linear ion-trap tandem MS/MS. The identified lipids included phosphatidylcholines (PCs), lyso-phosphatidylcholines (LPCs), phosphatidylethanolamines (PEs) and lyso-phosphatidylethanolamines (LPEs), phosphatidylserines (PSs), phosphatidylglycerols (PGs), and phosphatidylinositols (PIs). The presence of lysyl-phosphatidylglycerols (LyPGs) was identified as a key finding, marking a lipid class characteristic of *Gram-positive* bacterial membranes. This discovery highlights the role of viable bacteria in kefir and underscores its probiotic potential. The structural details of minor glycolipids (GLs) and glycosphingolipids (GSLs) were further elucidated, enriching the understanding of kefir’s lipid complexity. Fatty acyl (FA) composition was characterized using reversed-phase LC coupled with tandem MS. A mild epoxidation reaction with meta-chloroperoxybenzoic acid (m-CPBA) was performed to pinpoint double-bond positions in FAs. The dominant fatty acids were identified as C18:3, C18:2, C18:1, C18:0 (stearic acid), C16:0 (palmitic acid), and significant levels of C14:0 (myristic acid). Additionally, two isomers of FA 18:1 were distinguished: ∆9-cis (oleic acid) and ∆11-trans (vaccenic acid). These isomers were identified using diagnostic ion pairs, retention times, and accurate *m*/*z* values. This study provides an unprecedented level of detail on the lipid profile of kefir, shedding light on its complex composition and potential nutritional benefits.

## 1. Introduction

Kefir is a fermented milk, a carbonated and slightly alcoholic beverage, originating from the Caucasus and Tibet, traditionally produced using kefir grains. These grains consist of a unique and complex mixture of bacteria and yeasts that exist in a symbiotic relationship, working together to ferment milk [1]. The grains, which are gelatinous and resemble small cauliflower florets, are typically added to cow, goat, or sheep milk to initiate the fermentation process. Fermentation using selected starter cultures is recognized as an effective method to enhance food safety and nutritional/functional properties. It achieves this by eliminating microbial contamination, producing novel bioactive compounds or degrading anti-nutritional substances while enriching the food’s sensory and technological properties [2]. Given these advantages, kefir has garnered significant scientific interest due to its numerous health benefits. In vivo studies over the years have confirmed various health-promoting effects of kefir, including antioxidant [3], immunological [4], and antibacterial properties [5], as well as improved digestion and lactose tolerance [6]. Additional benefits include hypocholesterolemic [7], anti-inflammatory, anti-carcinogenic, anti-allergenic [8], and antihypertensive effects [9], and the control of plasma glucose levels.

Kefir has been recognized as one of the nine food trends by the Institute of Food Technologists, highlighting its growing appeal in the food industry. Its unique sensory characteristics, low lactose content, and functional health benefits make it stand out. The increasing awareness of probiotics and their positive effects on gut health have certainly played a role in their rising popularity. As more people seek functional foods with health benefits, kefir seems poised for continued growth, with projections estimating an annual market growth of over 6.3% from 2024 to 2032 [10].

The nutritional value of kefir varies depending on milk composition, the specific microorganisms in the grains, fermentation conditions (time and temperature), and storage [11,12]. Research into kefir’s beneficial components has largely focused on proteins, peptides [13,14], and vitamins [15]. However, the characterization of its lipophilic fraction, particularly triglycerides and phospholipids (PLs), remains comparatively underexplored [16,17,18]. Dietary PLs play a critical role in cellular membranes by delivering fatty acids (FAs) for incorporation into membrane structures. The composition of PLs directly impacts membrane properties such as fluidity and the formation of lipid rafts, underscoring the importance of characterizing kefir’s PL content [19].

Liquid chromatography (LC) coupled with mass spectrometry (MS) is widely considered the gold standard for lipid separation in food analysis, owing to its exceptional resolution and high repeatability. This technique outperforms other separation methods, particularly in the analysis of bioactive lipids, as demonstrated by recent studies in food such as lupins [20], strawberries [21], milk [22], fish [23], etc. Various high-performance (HP) LC techniques, including normal-phase (NP), reversed-phase (RP), and hydrophilic interaction liquid chromatography (HILIC), are commonly employed for lipid analysis. Among these, HILIC is especially favored for its ability to efficiently separate polar compounds through strong interactions with the stationary phase, making it particularly effective for analyzing hydrophilic lipids [24,25].

This study aimed to comprehensively analyze the lipid profile of kefir derived from bovine milk. HILIC coupled with electrospray ionization (ESI) in negative mode was employed, using high-resolution Fourier-transform orbital-trap MS (HR-FTMS) and linear ion-trap (LIT) tandem MS (HILIC-ESI-MS/MS). To evaluate the influence of microbiota on lipid composition, commercial kefirs produced using two industrial starter cultures were compared with each other and with milk. One kefir (sample 1) contained *L. lactis*, *L. cremoris*, *L. acidophilus*, *L. helveticus*, *S. thermophilus*, and *B. lactis*, while the other (sample 2) included *Streptococcus* sp., *Lactobacillus* sp., *Lactococcus* sp., *Leuconostoc* sp., and *B. lactis*. Also, fruit kefirs with the same starter’s cocktail were considered. The combination of accurate MS, data-dependent analysis (DDA), all-ion fragmentation (AIF), and targeted tandem MS enabled the identification of nearly 300 lipids, including PLs, lyso PLs (LPLs), glycosphingolipids, glycolipids, and amino-phospholipids like lysyl-phosphatidylglycerol (LyPG), a lipid specific to *Gram-positive* bacteria [26]. Negative-ion mode ESI provided more informative results compared to positive-ion mode. The ionization in negative-ion mode is likely mainly exploiting the easy proton loss from the phosphate and sulfonate groups, leading to the formation of the [M-H]^−^ adducts.

Chemical hydrolysis of the lipid extracts released major and minor FAs, which were analyzed by RPLC-ESI-MS. The results identified oleic, palmitic, stearic, linoleic, and myristic acids as the dominant fatty acyl chains. Furthermore, the use of meta-chloroperoxybenzoic acid (m-CPBA) to epoxidize the double bonds in unsaturated FAs, followed by tandem MS (MS/MS) analysis, allowed the localization of C=C positions along the fatty acyl chains. Diagnostic product ions generated during the fragmentation of epoxidized FAs (epo-FAs) provided detailed insights into the lipid structure [27,28,29].

## 2. Results and Discussion

### 2.1. Phospholipid Analysis of Kefir by HILIC-ESI-FTMS

**Figure 1:**
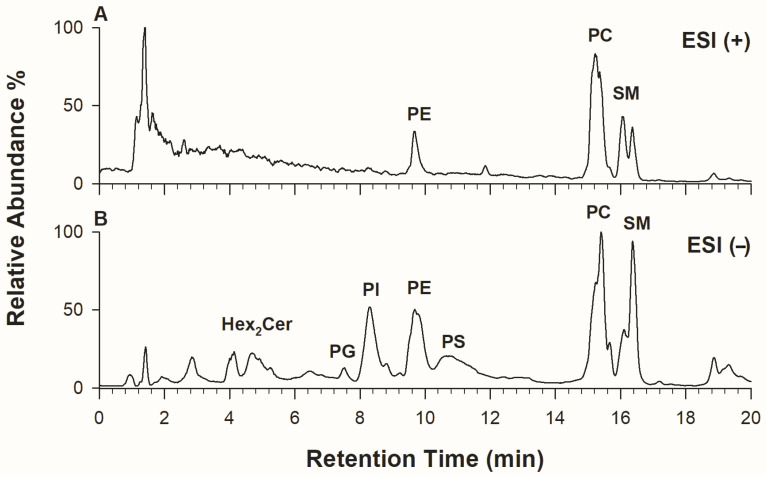
Representative total ion current (TIC) chromatograms by HILIC-ESI-HR-FTMS of a lipid extract from kefir sample 1, analyzed in both positive (**A**) and negative (**B**) ion modes. The abbreviations indicate the individual lipid classes separated during the analysis: Hex_2_Cer (Dihexosylcerebroside), PG (Phosphatidylglycerol), PI (Phosphatidylinositol), PE (Phosphatidylethanolamine), PS (Phosphatidylserine), PC (Phosphatidylcholine), and SM (Sphingomyelin).

As previously documented, HILIC simplifies the analysis of complex lipid mixtures by separating compounds based on the polarity of their head groups, resulting in chromatograms with distinct bands corresponding to each lipid class [30,31]. Figure 1 illustrates representative total ion current (TIC) chromatograms in negative (A) and positive (B) ion modes for a lipid extract derived from kefir (sample 1) fermented with *L. lactis*, *L. cremoris*, *L. acidophilus*, *L. helveticus*, *L. lactis*, *S. thermophilus*, and *B. lactis*. As anticipated, phosphatidylcholines (PCs), lysophosphatidylcholines (LPCs), and sphingomyelins (SMs) produced strong peak signals in positive-ion mode. However, the negative-ion mode proved more informative, as it enabled the effective ionization of a wider range of lipid classes. While SMs, PCs, and LPCs typically ionize as formate or acetate adducts, applying in-source-induced fragmentation (sid) at a collisional energy of 40 eV enhanced the generation of [M-H-CH_3_]^−^ ions [32], providing valuable insights into their regiochemistry. In contrast, anionic PLs such as phosphatidylinositols (PIs), phosphatidylethanolamines (PEs) and their lyso-forms (LPEs), phosphatidylserines (PSs), and phosphatidylglycerols (PGs) exhibited higher ionization yields in negative-ion mode, predominantly appearing as deprotonated forms. Additionally, less-abundant lipid classes, including cerebrosides (Hex_1_Cer, Hex_2_Cer, and Hex_3_Cer) and digalactosyldiglycerides (DGDGs), were identified through the formation of chloride or formate adducts [33,34].

All major PL classes eluted within 20 min, following the expected order of increasing headgroup polarity: PGs > PIs > PEs > PSs > LPEs > PCs > SMs > LPCs [35]. The lipid composition within each HILIC band was determined by averaging the MS spectra across the corresponding retention time window. Putative molecular formulas were proposed based on accurate FTMS *m*/*z* values, using the Online Lipid Calculator tool (with a tolerance of ±0.005 units). Only formulas with a relative error between experimental and theoretical *m*/*z* values of less than 5 ppm were considered valid.

To determine whether significant changes occur in the PL profile of milk after fermentation, the lipid composition of bovine milk (see Materials and Methods) was analyzed (Figure S1). Initial comparisons revealed that fermenting milk into kefir significantly alters the overall PL composition, particularly for PCs, PEs, PGs, PSs, PIs, and SMs. These findings align with recent studies on bovine milk, yogurt, and cheese [17,36]. To quantify these changes, we analyzed three extracted samples in triplicate for both milk and kefir. For each sample, we generated extracted ion current (EIC) profiles for each PL class, integrated the peak areas, and calculated the class-normalized relative abundance (%) across all detected species. These results, along with mass accuracy and retention times, are detailed in Table S1.

**Figure 2:**
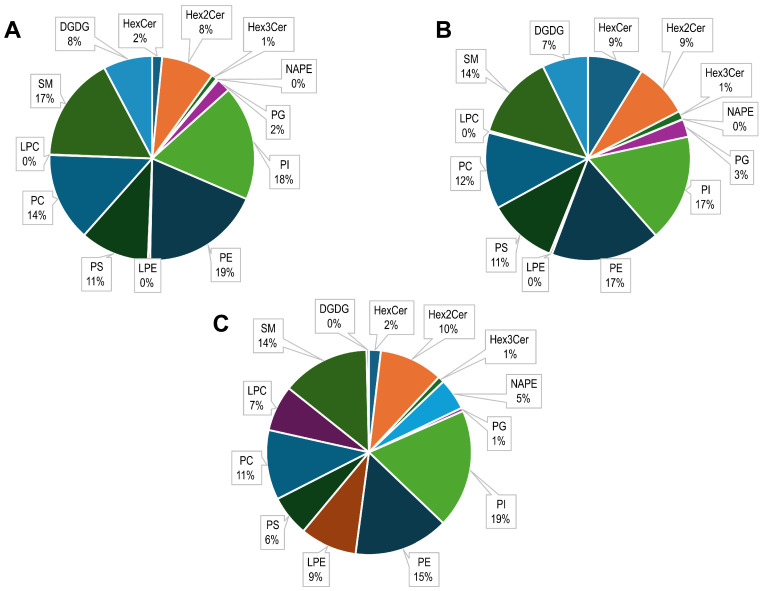
Phospholipid class distributions, as evaluated by HILIC-ESI-MS considering class-related peak areas, of kefir samples 1 and 2 (**A**,**B**), respectively, and milk (**C**).

Pie charts in Figure 2 illustrate the relative abundance of the major PL classes detected in kefir 1 (A), kefir 2 (B) and bovine milk (C). The charts highlight negligible differences between the two kefir samples and clear differences in lipid distribution between milk and both kefirs, underscoring the impact of fermentation and starter cultures on lipid composition. Key observations include: N-acyl phosphatidylethanolamines (NAPEs) were negligible in both kefir samples but more prominent in milk, consistent with its known role in dairy products; lyso forms were more abundant in milk, while glycolipids as DGDGs and hexosylceramides (HexCer) were significantly more prevalent in kefir samples; conventional PL classes (e.g., PCs, PEs, PSs, PIs, SMs) showed no significant differences between milk and kefir, likely due to variations in the origin of the milk, which strongly influences lipid composition; PG levels were notably higher in kefir, potentially linked to the presence of unique lipid species in these samples (vide infra). The increase in glycolipids is noteworthy, since a recent study analyzing the alteration of carbohydrates in kefir samples showed that when fermentation occurs, the composition of galactooligosaccharides changed due to the lactose conversion. This change in lactose content could be also correlated to the variation in glycosylated lipids [37].

### 2.2. Exploring Glycosphingolipids (GSLs) in Kefir by HILIC-ESI-FTMS

**Figure 3:**
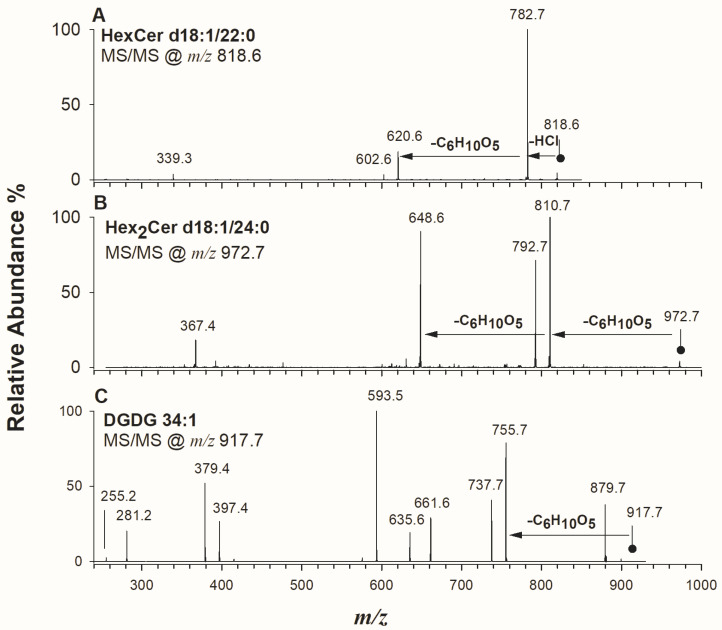
Tandem MS spectra for key lipid species identified in kefir samples: (**A**) HexCer d18:1/22:0, observed as a chlorinated adduct with an *m*/*z* value of 818.6; (**B**) Hex_2_Cer d18:1/24:0, detected as a deprotonated molecule at *m*/*z* 972.7, and (**C**) DGDG 34:1 as a deprotonated species at *m*/*z* 917.7.

A deeper analysis of the liquid chromatograms by HILIC-ESI-FTMS reveals subtle yet significant bands corresponding to GSLs at retention times of approximately 2, 4, and 6 min. These bands were attributed to mono-, di-, and tri-HexCer with various isomeric forms. Mass spectrometry analysis in negative-ion mode confirmed their ionization primarily as [M−H]^−^, [M+Cl]^−^, and [M+HCO_2_]^−^, providing crucial data for structural elucidation [38]. The fragmentation patterns in negative polarity offered complete information on the GSL head group, sphingoid base (SB), and fatty acid composition. For instance, the MS/MS spectrum of a HexCer species at *m*/*z* 818.6 (Figure 3A) revealed a pronounced signal at *m*/*z* 782.7, arising from the loss of 36 Da (-HCl), consistent with a chloride adduct. Accurate mass measurements, as summarized in Table S1, supported this assignment. Key diagnostic ions included: neutral loss of 162.0 Da, corresponding to a sugar moiety, yielding the ion at *m*/*z* 620.6; water loss from the sphingoid backbone (*m*/*z* 602.6), which introduced an additional double bond; carboxylate anion of C22:0 fatty acid at *m*/*z* 339.3, resulting from the rearrangement of the acyl chain, pinpointing the specific fatty acid substituent [39]. Thus, the ion at *m*/*z* 818.6 was confidently identified as HexCer d18:1/22:0, ionized as a chloride adduct. Similarly, the MS/MS spectrum of the ion at *m*/*z* 972.7 (Figure 3B) indicated a Hex_2_Cer. Sequential losses of 162.0 Da confirmed the presence of two hexose units. The analysis of diagnostic “a” and “b” ions allowed the identification of SB and fatty acid chains as Hex_2_Cer d18:1/24:0 [33,38]. Lastly, Figure 3C depicts the MS/MS spectrum of the glycolipid DGDG 34:1. Here, two successive losses of 162 Da led to the formation of a deprotonated diacylglycerol (DAG 34:1) ion at *m*/*z* 593.5. Additional ions at *m*/*z* 661.6 and *m*/*z* 635.6 corresponded to the cleavage of esterified acyl chains (16:0 and 18:1, respectively), with characteristic carboxylate signals at *m*/*z* 255.2 and *m*/*z* 281.2.

GSLs are structurally complex lipids integral to biological membranes, including the milk fat globule membrane (MFGM) found in mammalian milk. They play pivotal roles in membrane stability, biological activity, and cell signaling. The presence of GSLs in milk and kefir, along with their varying distributions, underscores their functional importance across different lactation stages and mammalian species. Several factors contribute to variations in the relative abundance of PLs and GSLs in milk and kefir, such as animal breed, lactation stage, dietary influences, type of kefir grains, and fermentation duration. These variables can significantly influence the lipid profile, reflecting their critical roles in the structural and functional properties of the beverage. Our focus on identifying distinctive lipids associated with viable microorganisms in kefir uncovered species such as lysyl-phosphatidylglycerols, which are markers of *Gram-positive* bacteria (see next paragraph). This finding not only highlights the intricate microbiota–lipid interactions in kefir but also opens avenues for future research into bioactive lipids and their implications for health and nutrition [40,41,42].

### 2.3. Lysyl-Phosphatidylglycerols in Kefir Beverage

**Figure 4:**
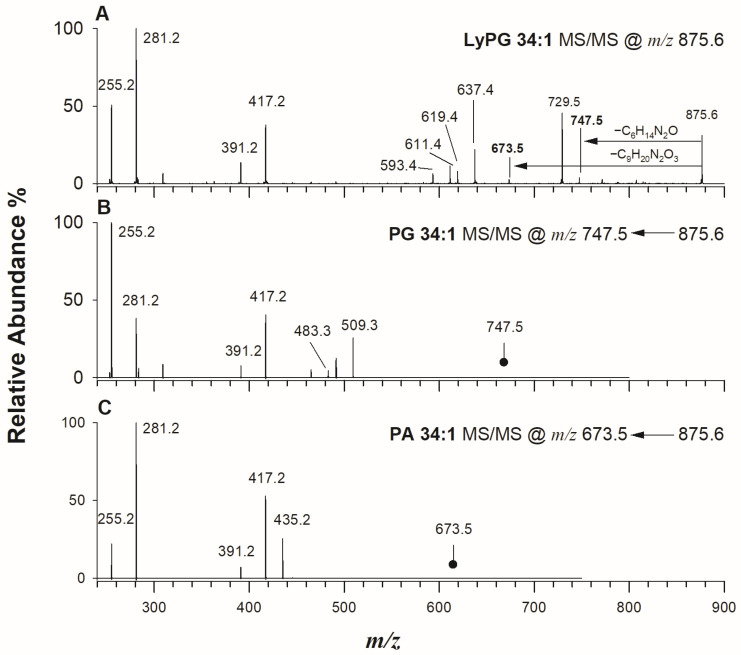
(**A**) Tandem MS spectrum at *m*/*z* 875.6 of lysyl-PG 34:1. MS^3^ spectra of ions at *m*/*z* 747.5 (**B**) and *m*/*z* 673.5 (**C**) isolated from precursor at *m*/*z* 875.6. All species were detected as deprotonated molecules. PG and PA stand for phosphatidylglycerols and phosphatidic acids, respectively.

**Scheme 1:**
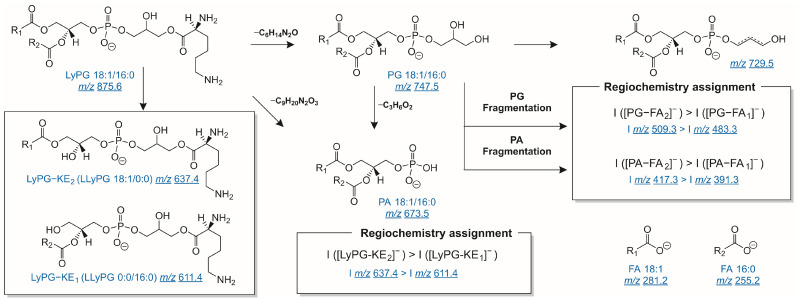
Proposed fragmentation pathway of lysyl-phosphatidylglycerols. The ions specifically generated from the fragmentation of LyPG 18:1/16:0 at *m*/*z* 875.6 of Figure 4 are highlighted in blue.

LyPGs are well-characterized membrane lipids found in various *Gram-positive* bacteria, such as *S. aureus*, *B. subtilis*, *L. plantarum*, and *Lactobacillus* species [26,31]. Given the microbial composition of kefir, we anticipated the presence of this lipid class in kefir but not in unfermented milk. Indeed, upon extracting the ion current at *m*/*z* 875.613, corresponding to the accurate mass-to-charge ratio of LyPG 34:1 [43,44], a chromatographic peak at approximately 15 min in both kefir samples was observed. This intriguing finding prompted further investigation using tandem mass spectrometry with a linear ion trap to elucidate the regiochemical details of LyPG molecules. Upon collision-induced dissociation (CID) of the ion at *m*/*z* 875.6, the resulting product ion spectrum (Figure 4A) displayed several diagnostic peaks. Key fragments included *m*/*z* 747.5, generated by the loss of the lysyl residue, corresponding to a PG 34:1; *m*/*z* 729.5, arising from the dehydration of a PG 34:1; *m*/*z* 673.5, corresponding to the formation of a PA 34:1; *m*/*z* 281.2 and *m*/*z* 255.2, representing the fatty acid anions C18:1 and C16:0 (palmitate), respectively. This fragmentation pattern suggests that the LyPG species in question can be tentatively assigned as LyPG 18:1_16:0. All the ions detected in Figure 4 are reported in Scheme 1.

The regiochemical assignment was confirmed by CID-MS³ experiments, analyzing fragments of ions at *m*/*z* 747.5 (PG 34:1) and *m*/*z* 673.5 (PA 34:1). The resulting product ions were interpreted following the rules previously established by Hsu and Turk [45,46]. In the MS³ spectrum of negatively charged PG 34:1 (*m*/*z* 747.5; Figure 4B), diagnostic fragment ions provided key insights. The first group of product ions is made up of signals at *m*/*z* 255.2 (palmitate anion, 16:0), 491.3 (neutral loss of FA 16:0), and 509.3 (neutral loss of a FA 16:0 as ketene), whereas the second group includes peaks at *m*/*z* 281.2 (most probably oleate anion, 18:1, vide infra), 465.3 (neutral loss of FA 18:1), and 483.3 (neutral loss of a FA 18:1 loss as ketene). Importantly, the relative intensities of these fragment ions demonstrated preferential loss of the sn-2 fatty acid (C16:0) over the sn-1 chain, consistent with previously established fragmentation rules [45]. Similar analysis of PA 34:1 (*m*/*z* 673.5; Figure 4C) supported the same regiochemical assignment, confirming the sn-2 position for C16:0 and the sn-1 position for C18:1. These findings established the structure as LyPG 18:1/16:0. Further evidence was gleaned from the MS/MS spectrum of intact LyPG 18:1/16:0 (Figure 4A), where the relative intensities of ions at *m*/*z* 637.4 ([M-H-KE 16:0]^−^) and *m*/*z* 611.4 ([M-H-KE 18:1]^−^) corroborated the regiochemical assignment [46]. This systematic approach was applied to all LyPG species detected in kefir samples, with results summarized in Table 1. Additional MS/MS spectra for other LyPG species are provided in Figure S2.

Using higher-energy collision dissociation (HCD) in an Orbitrap instrument revealed a highly informative fragmentation pattern. At 35 eV, a prominent base peak at *m*/*z* 145.098, corresponding to deprotonated lysine ([C6H13N2O2]^−^), emerged (Figure S3). This lysine-derived fragment could serve as a diagnostic marker for LyPGs in all-ion fragmentation scans, providing a robust means of confirming the elution profile of this typical lipid class or reinforcing its identification.

This study represents the first identification of lysyl-phosphatidylglycerols in kefir, highlighting their diagnostic value as markers for *Gram-positive* bacteria in fermented beverages. Beyond offering structural insights, the presence of LyPGs unveils promising research opportunities, such as their functional role in microbial membrane dynamics, stress tolerance, and the probiotic traits of kefir-associated microbes. The biomarker potential of LyPGs also presents practical applications, such as evaluating fermentation quality and microbial viability in dairy products. Comparative investigations into LyPG distribution across different kefir types, fermentation conditions, and milk sources could shed light on how lipid profiles relate to the health-promoting properties of kefir. These findings pave the way for novel explorations into kefir’s nutritional benefits and its intricate relationship with probiotic bacterial activity, further enriching our understanding of this functional food.

### 2.4. RPLC-ESI-MS/MS of Linked Fatty Acyl Chains upon Chemical Hydrolysis

**Figure 5:**
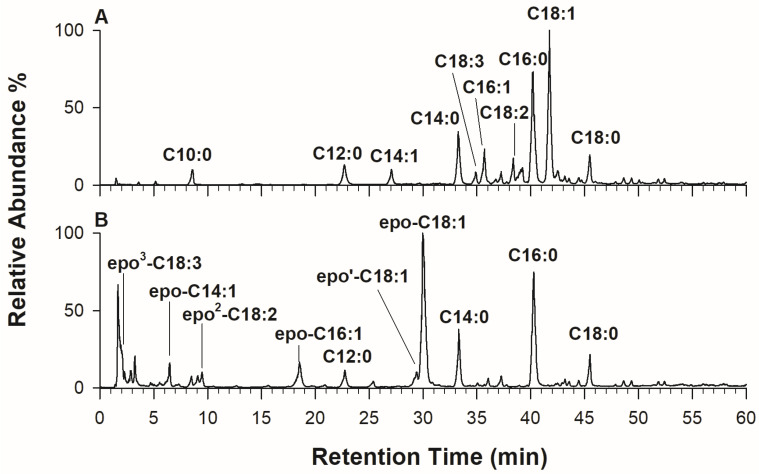
Total ion current chromatograms by RPLC-ESI(-)-HR-FTMS of a kefir lipid extract (sample 2) analyzed in two steps: (**A**) following hydrolysis of the extracted lipids and (**B**) after subsequent epoxidation of the same sample.

To investigate potential differences in fatty acyl chain composition, the types of linked fatty acids in kefir were analyzed using chemical hydrolysis followed by an epoxidation reaction. The hydrolyzed lipid extract was examined by RPLC-ESI(−)-HRFTMS before (Figure 5A) and after epoxidation (Figure 5B), with each FA identified as a deprotonated molecule, [M−H]^−^. Peak identification was achieved by matching retention times with a standard FA solution [47] and collecting accurate *m*/*z* ratios. Peaks were labeled using the conventional C:D nomenclature, where C represents the number of carbon atoms and D denotes the number of double bonds. In RPLC, FA separation is influenced by chain length and degree of unsaturation. For instance, C18:0 was observed to elute later (45.5 min) than C18:1 (41.7 min) and C16:0 (40.2 min), as the double bond effectively reduces the chain length by approximately two carbon units. Consistent with prior research by Månsson [48] on milk, FAs ranging from 8 to 28 carbon atoms were detected (shorter FAs were not detectable with this setup), including odd-chain FAs (e.g., 15:0, 17:0, 19:0) and hydroxylated FAs. The presence of odd- and branched-chain FAs in bovine milk, attributed to rumen microbiota activity, has been previously reported [48]. These unique FAs, influenced by factors such as cow diet and rumen environment, are known to contribute not only to the nutritional profile but also to the sensory and functional properties of milk and kefir, impacting flavor and texture. It is also suggested that their presence in kefir may arise from microbial membrane disruption, particularly from Lactobacilli or other kefir-associated strains.

**Figure 6:**
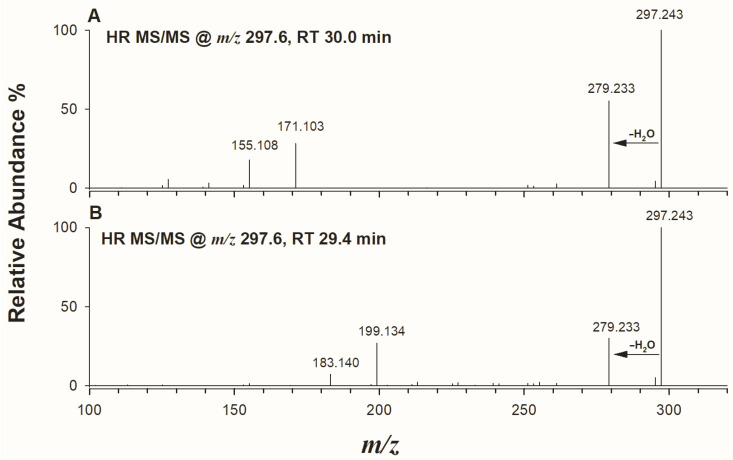
MS/MS spectrum of deprotonated-epoxidated (**A**) FA 18:1, ∆9 and (**B**) FA 18:1, ∆11, which elute at 30.0 and 29.4 min, respectively (see Figure 5).

While saturated FAs were confidently identified based on their accurate *m*/*z* ratios and retention times, the determination of double-bond positions in unsaturated FAs required further investigation [49]. To this end, mild epoxidation was performed on a hydrolyzed sample, enabling the separation of oxidized FAs (Figure 5B). As a case study, FA 18:1 was examined (Figure 6). The value at *m*/*z* 281.2481, corresponding to deprotonated FA 18:1 ([C18H34O2-H]^−^), was observed to generate an epoxidized species ([C18H34O3-H]^−^), monoisotopic mass 297.2435) detected at 30.0 min (Figure 5B). Tandem MS analysis revealed diagnostic ion pairs at *m*/*z* 155.108/171.103 (Figure 6A), confirming the presence of FA 18:1 ∆9 as the predominant species. A second chromatographic peak at 29.4 min, corresponding to another epoxidized FA 18:1, was identified as FA 18:1 ∆11, based on the product ions at *m*/*z* 183.140 and 199.134 (Figure 6B). The identification of both of these peaks as FA 18:1 ∆9-cis (oleic acid) and 18:1 ∆11-trans (vaccenic acid) [50] was based on their retention times matching those of available standards within ±0.1 min and accurate *m*/*z* values within less than 5 ppm of the theoretical values. Despite these findings, the overall distribution of FAs in kefir was found to closely resemble that of cow milk, suggesting that fermentation does not significantly alter fatty acyl chain composition. This insight raises intriguing questions about the stability of lipid profiles during fermentation and their potential role in the functional and sensory attributes of kefir.

## 3. Materials and Methods

### 3.1. Chemicals and Commercial Samples

LC-MS-grade water (H_2_O), methanol (MeOH), acetonitrile (ACN), chloroform (CHCl_3_), formic acid, ammonium formate, hydrochloric acid (HCl), and meta-chloroperoxybenzoic acid (*m*-CPBA), were obtained from Merck (Milan, Italy). Standard lipids were obtained from Spectra 2000 SRL (Rome, Italy). A calibrating solution containing caffeine, methionine-arginine-phenylalanine-alanine peptide and Ultramark, a mixture of fluorinated phosphazines, was purchased from Thermo Scientific (Waltham, MA, USA). Standard FAs were obtained by the chemical hydrolysis of a 37-Component FAME Mix on SP™-2560 (Merck, Milan). EquiSPLASH^®^ LIPIDOMIX^®^ solution containing 13 deuterated lipid standards at a concentration of 100 µg/mL each (PC 15:0/18:1(d7), LPC 18:1(d7), PE 15:0/18:1(d7), LPE 18:1(d7), PG 15:0/18:1(d7), PI 15:0/18:1(d7), PS 15:0/18:1(d7), TG 15:0/18:1(d7)/15:0, DG 15:0/18:1(d7), MG 18:1(d7), CE 18:1(d7), SM 18:1/18:1(d9), and ceramide 15(d7)) were obtained from Merck (Milan, Italy). 

Commercial kefir and milk were sourced from local supermarkets. Specifically, five types of kefir samples were analyzed: two plain (discussed in detail in the text) and three flavored varieties (strawberry, blueberry, and apricot). The fruit kefirs were selected to contain the same strains of bacteria as the plain kefir. For the milk samples, five commercial varieties were purchased, and an additional sample was created by combining equal aliquots from each milk. Each sample was extracted in duplicate and analyzed in triplicate.

### 3.2. Sample Preparation

#### 3.2.1. Lipid Extraction

Following the Bligh &Dyer protocol [51], 3 mL of MeOH/ CHCl_3_ (2:1, *v*/*v*) was added to 800 µL of kefir or milk. Then, 1 mL of CHCl_3_ was added, and the mixture was vortexed for 30 s. Finally, 1 mL of H_2_O was added, and the solution was shaken before being centrifuged for 10 min at 2000 rpm. The lower phase containing lipids was dried under nitrogen flow; the residue was dissolved in 100 µL of MeOH/CHCl_3_ (1:1, *v*:*v*) and then analyzed by LC-MS, injecting 5 µL of extract.

#### 3.2.2. Chemical Hydrolysis of Linked Fatty Acyl Chains

The dried product resulting from Bligh&Dyer extraction was taken up in 1 mL of HCl 0.5 M in ACN: H_2_O (9:1, *v*:*v*) and left at 100 °C for 15 min. Then, 1 mL of CHCl_3_ and 1 mL of H_2_O were added, and the mixture was centrifuged for 15 min at 3000× *g*. The organic phase was collected, 1 mL of H_2_O was added, vigorously mixed, and then centrifuged again for 15 min at 3000× *g*. The organic phase was recovered, dried under nitrogen, and dissolved in 1 mL of CHCl_3_:/MeOH (1: 1, *v*:*v*) for successive RPLC analysis.

#### 3.2.3. Olefine Epoxidation

The epoxidation of hydrolyzed FA was carried out by modifying the protocol reported by Coniglio et al. [21]. Briefly, the lipid extract was dissolved in 500 µL of a chloroform solution containing *m*-CPBA at 20 µg/µL and left at room temperature for 15 min; then, 2 mL of ACN/ H_2_O (1:1, *v*:*v*) was added to quench the reaction, and, finally, the sample was dried under a gentle nitrogen (N_2_) flow and dissolved in 100 µL of the mobile phase initial composition, to be subjected to LC-ESI-MS analyses.

### 3.3. LC-ESI-MS Instrumentation and Operating Conditions

HILIC-ESI-MS and RPLC-ESI-MS measurements were performed using an LC-MS apparatus consisting of a UHPLC system Ultimate 3000 and a hybrid Q-Exactive mass spectrometer (Thermo Scientific, Waltham, MA, USA), equipped with a heated electrospray ionization (HESI) source and a higher collisional energy dissociation (HCD) cell for tandem MS analyses or a Velos Pro mass spectrometer (Thermo Scientific) equipped with a linear ion trap analyzer.

HILIC separations were run at 25 °C on a narrow-bore column (150 mm × 2.1 mm ID, 2.7 μm particle size) equipped with a security guard cartridge (5 mm × 2.1 mm ID), both Ascentis Express HILIC (Supelco, Bellefonte, PA, USA), using a flow rate of 300 µL/min. The adjusted binary elution program, based on H_2_O and 2.5 mmol ammonium formate (solvent A) and ACN (solvent B), both containing 0.1% (*v*/*v*) of formic acid, was adopted: 0–5 min, linear gradient from 97 to 88% solvent B; 5–10 min, isocratic at 88% solvent B; 10–11 min, linear gradient from 88 to 81% solvent B; 11–20 min, linear gradient from 81 to 70% solvent B; 20–22 min, linear gradient from 70 to 50% solvent B; 22–28 isocratic at 50% solvent B; 28–30 min, return to the initial composition, followed by a 5 min equilibration time.

RPLC separations were based on an Ascentis Express C18 column also packed with yescore-shell particles (150 mm × 2.1 mm ID, 2.7 µm particle size, 1.7 µm core size) and equipped with an Ascentis Express C18 (5 mm × 2.1 mm ID) security guard cartridge (Supelco, Bellefonte, PA, USA) at 40 °C, using a flow rate of 200 µL/min. RPLC runs were performed using a binary gradient based on H_2_O (eluent A) and MeOH (eluent B), both containing 2.5 mM ammonium acetate. The gradient elution program was the following: 0–10 min isocratic at 60% (*v*/*v*) solvent B; 10–50 min linear from 60% to 100% (*v*/*v*) solvent B; 50–58 min isocratic at 100% (*v*/*v*) solvent B; 58–63 min linear from 100% to 60% (*v*/*v*) solvent B, followed by 12 min equilibration time.

The column effluent was transferred into the mass spectrometer through the HESI source. The main ESI and ion optic parameters were the following: sheath gas flow rate, 35 arbitrary units (a.u.); auxiliary gas flow rate, 15 a.u.; spray voltage, 3.5 kV (positive) and −2.5 kV (negative); capillary temperature, 320 °C; S-lens radio frequency level, 100 a.u. Negative and positive MS full-scan spectra were acquired in the *m*/*z* range 130–2000, after setting a mass resolving power of 140,000 (at *m*/*z* 200). Besides accurate masses, LC-MS runs using DDA and AIF acquisitions were performed to confirm the lipid species. MS/MS measurements were run using a 1 *m*/*z* unit-wide window, a resolving power of 70,000 (at *m*/*z* 200), a fill time of 100 ms, and AGC of 2 × 10^5^. Furthermore, targeted MS measurements were performed in parallel using a medium-resolving-power Velos Pro mass spectrometer where the double-stage linear ion trap mass analyzer works in a low-energy CID regime, being complementary to HCD to confirm some doubtful attributions or to perform MS3 analyses when necessary. Only the S-lens radio frequency level, lowered to 60 arbitrary units, was modified among HESI and ion optic parameters when using the Velos Pro spectrometer. The collision energy was varied according to the ion of interest, from 35 to 45% (in this case, a 400% value corresponds to a 100 V excitation voltage) using a 1 *m*/*z* unit-wide isolation window centered on *m*/*z* ratio.

### 3.4. Preliminary Identification of Lipids by the Online Lipid Calculator

A preliminary identification of lipids extracted from kefir was performed using the Online Lipid Calculator, available freely at the following address: www.mslipidomics.info/lipid-calc/ (accessed on 17 October 2024). Given a certain *m*/*z* value and tolerance, the software retrieves the possible candidate structures among several lipid classes. The software calculates also different positively or negatively charged species relevant to the *m*/*z* values in the input. In the present study, the input values correspond to accurate *m*/*z* ratios retrieved from MS spectra obtained with the Q-Exactive spectrometer.

## 4. Conclusions

The comprehensive characterization of polar lipids in kefir was successfully achieved using LC-ESI-MS, leading to the identification of nearly 300 lipid species. By integrating semi-untargeted and targeted approaches, we obtained a detailed lipid profile of the kefir beverage. Notably, Lysyl-PG emerged as a key lipid marker linked to the presence of viable bacteria in kefir, as it was absent in non-fermented milk. Additionally, the analysis of bound fatty acids revealed the presence of odd-chain fatty acids, alongside dominant species such as C18:1 (both oleic and vaccenic acid), C16:0, C18:0, and C18:2. These findings underscore the importance of lipid studies in understanding how variations in feeding practices or fermentation processes influence milk quality. They also provide valuable insights into the metabolic activities occurring within the rumen.

## Figures and Tables

**Table 1:** Lysyl-phosphatidylglycerols (LyPGs) identified in kefir samples by HILIC-ESI(-)-HRMS in negative-ion mode as deprotonated species ([M-H]−). Accurate masses with errors in ppm, sum formula, and lipid species are reported.

LyPG	Accurate *m*/*z* Value	Theoretical *m*/*z* Value	D (ppm)	Empirical Formula (M)	Regiochemical Assignment
32:0	849.597	849.598	1.8	C_44_H_87_N_2_O_11_P	16:0/16:0
32:1	847.581	847.582	1.7	C_44_H_85_N_2_O_11_P	16:1/16:0
34:1	875.612	875.613	0.8	C_46_H_89_N_2_O_11_P	18:1/16:0
34:2	873.596	873.598	2.1	C_46_H_87_N_2_O_11_P	18:1/16:1 and 18:2/16:0
34:3	871.580	871.582	2.4	C_46_H_85_N_2_O_11_P	18:3/16:0
35:1	889.627	889.629	2.0	C_47_H_91_N_2_O_11_P	19:1/16:0 and 18:1_17:0
36:1	903.644	903.645	1.0	C_48_H_93_N_2_O_11_P	20:1/16:0 and 18:1/18:0
36:2	901.628	901.629	1.2	C_48_H_91_N_2_O_11_P	18:1/18:1, 20:1/16:1 and 20:2_16:0
37:2	915.643	915.645	2.5	C_49_H_93_N_2_O_11_P	19:1/18:1
38:1	931.675	931.676	0.7	C_50_H_97_N_2_O_11_P	20:0/18:1
38:2	929.660	929.660	0.2	C_50_H_95_N_2_O_11_P	20:1/18:1
38:3	927.642	927.645	3.8	C_50_H_93_N_2_O_11_P	20:1/18:2
40:2	957.689	957.691	2.1	C_52_H_99_N_2_O_11_P	20:1/20:1 and 18:1_22:1

## Data Availability

The raw data supporting the conclusions of this article will be made available by the authors upon request.

## Supplementary Materials

The following supporting information can be downloaded at: https://www.mdpi.com/article/10.3390/ijms26031120/s1.
