# Supplementary material for: Complete Polar Lipid Profile of Kefir Beverage by Hydrophilic Interaction Liquid Chromatography with HRMS and Tandem Mass Spectrometry
Source: Int J Mol Sci. 2025 Jan 28;26(3):1120. doi: 10.3390/ijms26031120 (PMC11818909; doi:10.3390/ijms26031120)
Supplement: Supplementary file 1 [file ijms-26-01120-s001.zip › ijms-3440743-supplementary.pdf]

Article

Complete Polar Lipid Profile of Kefir Beverage by Hydrophilic Interaction Liquid Chromatography with HRMS and Tandem Mass Spectrometry

Giovanni Ventura [1,2], Mariachiara Bianco [1,2], Ilario Losito [1,2], Tommaso R. I. Cataldi [1,2] and Cosima D. Calvano [1,2,*]

[1] Dipartimento di Chimica; Università degli Studi di Bari Aldo Moro, via Orabona 4, 70126 Bari, Italy; giovanni.ventura@uniba.it (G.V.); mariachiara.bianco@uniba.it (M.B.); ilario.losito@uniba.it (I.L.); tommaso.cataldi@uniba.it (T.R.I.C.)
[2] Centro Interdipartimentale SMART, Università degli Studi di Bari Aldo Moro, via Orabona 4, 70126 Bari, Italy
* Correspondence: cosimadamiana.calvano@uniba.it

Table S1. List of the identified lipid species by HILIC-ESI-MS/MS in negative ion mode as deprotonated molecules. Adduct observed, accurate masses with errors in ppm, retention time (RT), and relative % in three samples are reported.

n	Species	Adduct	*m/z*	RT (min)	Δ (ppm)	Kefir 1	Kefir 2	Milk
**1**	HexCer 32:1;2	+Cl-	706.502	1.48	1.7	4.1	0.5	2.6
**2**	HexCer 34:1;2	-H+	698.558	2.17	0.1	2.2	0.9	7.3
		+Cl-	734.534	1.42	0.4	22.2	4.2	18.7
**3**	HexCer 34:2;2	-H+	696.542	2.30	0.0	0.0	1.3	0.1
		+Cl-	732.519	1.53	-0.6	0.4	8.6	0.5
**4**	HexCer 34:2;3	-H+	712.538	2.45	-1.3	0.1	16.6	0.1
		+Cl-	748.514	1.29	-0.7	1.0	44.3	1.1
**5**	HexCer 36:1;2	+Cl-	762.564	2.14	2.3	0.8	0.1	1.1
**6**	HexCer 38:1;2	-H+	754.620	2.09	0.8	0.4	0.2	1.6
		+Cl-	790.596	1.45	0.6	5.5	1.1	4.7
**7**	HexCer 40:1;2	+HCOO-	828.657	2.02	0.1	0.7	0.7	2.6
		-H+	782.651	2.06	1.0	1.1	0.5	4.7
		+Cl-	818.628	1.41	0.9	17.7	5.3	15.1
**8**	HexCer 40:1;3	-H+	798.647	2.41	-0.6	0.8	0.4	1.3
**9**	HexCer 41:1;2	-H+	796.666	2.04	1.7	0.9	0.5	3.8
		+Cl-	832.644	1.36	0.1	15.2	5.2	12.0
**10**	HexCer 41:1;3	-H+	812.662	2.20	-0.2	0.7	0.3	1.3
		+Cl-	848.643	2.17	-5.0	5.7	1.7	4.3
**11**	HexCer 42:1;2	+HCOO-	856.687	2.03	2.0	0.3	0.3	1.1
		-H+	810.682	2.01	1.3	0.6	0.3	2.3
		+Cl-	846.658	2.04	1.9	11.1	3.9	8.0
**12**	HexCer 42:1;3	-H+	826.677	2.18	1.1	0.6	0.3	1.2
		+Cl-	862.654	1.55	0.3	4.6	1.6	3.1
**13**	HexCer 42:2;2	+Cl-	844.639	1.36	5.0	2.0	0.7	1.3
**14**	HexCer 43:2;2	+Cl-	858.668	2.24	-5.0	1.3	0.4	0.3
**1**	Hex2Cer 32:0;2	+Cl-	870.569	5.77	3.2	0.5	0.4	0.9
**2**	Hex2Cer 32:1;2	+Cl-	868.556	6.02	0.3	1.0	0.7	0.9
**3**	Hex2Cer 32:3;3	-H+	844.533	5.57	5.0	0.1	0.0	6.8
**4**	Hex2Cer 33:1;2	-H+	846.595	5.96	0.3	0.9	0.9	0.6
		+Cl-	882.570	5.74	2.0	0.6	0.5	0.4
**5**	Hex2Cer 34:1;2	-H+	860.609	5.66	1.3	8.7	10.4	7.6
		+Cl-	896.587	5.92	-0.1	6.6	5.1	5.5
		+HCOO-	906.614	5.68	2.0	0.9	0.9	0.6
**6**	Hex2Cer 35:1;2	-H+	874.626	5.86	0.7	0.5	0.6	0.9
**7**	Hex2Cer 36:0;2	-H+	890.657	5.52	0.9	0.4	0.4	0.4
**8**	Hex2Cer 36:1;2	-H+	888.642	5.81	0.1	0.8	1.0	1.2
		+Cl-	924.617	5.86	1.2	0.7	0.4	0.9
**9**	Hex2Cer 36:3;3	+Cl-	936.577	4.36	5.0	0.0	1.3	0.0
**10**	Hex2Cer 37:1;2	-H+	902.657	5.76	0.2	0.3	0.4	0.5
**11**	Hex2Cer 38:0;2	-H+	918.688	5.43	0.6	2.7	3.1	2.1
		+HCOO-	964.693	5.43	1.7	0.5	0.5	0.4

		+Cl-	954.666	5.47	-0.8	0.9	0.2	1.3
**12**	Hex2Cer 38:1;2	-H+	916.672	5.43	1.4	4.3	4.5	3.5
		+Cl-	952.649	5.74	0.5	2.2	0.9	2.5
		+HCOO-	962.681	5.71	-3.0	0.7	0.7	0.6
**13**	Hex2Cer 38:2;2	+Cl-	950.642	5.92	-5.0	0.4	0.3	0.1
**14**	Hex2Cer 39:0;2	-H+	932.705	5.63	-0.9	2.4	2.7	1.5
		+HCOO-	978.704	5.38	5.0	0.5	0.5	0.1
		+Cl-	968.682	5.43	-0.6	0.6	0.2	0.9
**15**	Hex2Cer 39:1;2	-H+	930.688	5.38	1.1	5.6	5.8	3.7
		+HCOO-	976.692	5.38	1.9	1.1	1.1	0.7
		+Cl-	966.665	4.94	0.6	1.7	0.4	2.6
**16**	Hex2Cer 40:0;2	-H+	946.717	5.6	3.1	2.8	3.2	2.0
		+HCOO-	992.718	5.35	5.0	0.7	0.8	0.1
**17**	Hex2Cer 40:1;2	-H+	944.704	5.61	0.4	12.0	13.1	9.0
		+HCOO-	990.709	5.6	0.6	3.0	3.1	1.9
		+Cl-	980.68	4.89	0.8	3.4	1.1	6.9
**18**	Hex2Cer 40:2;2	-H+	942.688	5.6	0.4	1.1	1.0	0.6
**19**	Hex2Cer 41:1;2	-H+	958.718	5.3	2.0	10.7	11.7	7.2
		+HCOO-	1004.723	5.31	2.6	2.8	3.0	1.7
		+Cl-	994.695	4.84	1.4	2.5	1.2	5.5
**20**	Hex2Cer 41:2;2	-H+	956.704	5.54	0.7	1.7	1.6	0.8
**21**	Hex2Cer 42:0;2	-H+	974.756	6.9	-4.5	0.7	1.2	0.8
**22**	Hex2Cer 42:1;2	-H+	972.735	5.48	0.7	7.3	8.8	5.0
		+HCOO-	1018.738	5.25	2.7	2.2	2.7	1.4
		+Cl-	1008.711	4.77	1.6	0.2	0.2	3.6
**23**	Hex2Cer 42:2;2	-H+	970.718	5.25	2.3	1.7	2.0	1.0
		+HCOO-	1016.723	5.27	2.7	0.5	0.5	0.2
**24**	Hex2Cer 43:0;3	-H+	1004.769	6.89	-5.0	0.0	0.0	3.8
**25**	Hex2Cer 43:1;2	-H+	986.750	5.45	1.1	1.1	1.2	1.0
**1**	Hex3Cer 30:0;2	+Cl-	1004.592	9.95	0.8	3.6	2.8	3.4
**2**	Hex3Cer 30:1;2	+Cl-	1002.577	9.89	0.2	1.7	1.2	1.5
**3**	Hex3Cer 31:0;2	+Cl-	1018.607	9.93	2.0	0.9	0.8	0.9
**4**	Hex3Cer 32:0;2	+Cl-	1032.622	9.91	2.7	3.4	2.7	3.5
**5**	Hex3Cer 32:1;2	+Cl-	1030.607	9.89	1.3	6.2	5.0	5.1
**6**	Hex3Cer 32:2;2	+Cl-	1028.594	9.86	-0.5	1.5	1.4	1.6
**7**	Hex3Cer 33:0;2	+Cl-	1046.636	9.88	3.6	0.6	0.5	0.7
**8**	Hex3Cer 33:1;2	+Cl-	1044.623	9.86	1.7	1.4	1.2	1.2
**9**	Hex3Cer 34:1;2	+Cl-	1058.638	9.85	1.7	16.6	16.1	14.7
**10**	Hex3Cer 34:2;2	+Cl-	1056.623	9.84	1.5	8.7	8.6	9.1
**11**	Hex3Cer 34:3;2	+Cl-	1054.608	9.84	0.2	2.6	6.4	2.2
**11**		+HCOO-	1064.628	9.48	5.0	1.7	2.7	5.8
**12**	Hex3Cer 35:1;2	+Cl-	1072.653	9.88	2.6	1.4	1.4	2.1
**13**	Hex3Cer 35:2;2	+Cl-	1070.637	9.17	3.2	0.0	0.1	0.8
**14**	Hex3Cer 36:1;2	+Cl-	1086.666	9.78	5.0	10.8	12.5	9.9
**15**	Hex3Cer 36:2;2	+Cl-	1084.653	9.68	2.5	27.2	22.0	22.6
**16**	Hex3Cer 36:3;2	+Cl-	1082.637	9.68	2.8	8.6	9.4	8.6
**17**	Hex3Cer 37:2;2	+Cl-	1098.668	9.08	3.3	0.2	0.1	1.5
**18**	Hex3Cer 38:1;2	+Cl-	1114.697	9.58	4.9	0.4	0.8	0.3
**19**	Hex3Cer 38:2;2	+Cl-	1112.683	9.58	3.2	0.7	1.2	0.8
**20**	Hex3Cer 38:3;2	+Cl-	1110.665	9.56	5.0	1.7	3.0	3.6
**1**	DGDG 28:0	+Cl-	871.518	3.83	1.1	0.7	0.4	0.0
**2**	DGDG 30:0	+Cl-	899.550	3.72	0.5	3.1	4.9	9.5
**3**	DGDG 30:1	+Cl-	897.533	1.52	1.8	0.9	0.8	0.0
**4**	DGDG 32:0	+Cl-	927.580	3.33	1.6	2.8	3.8	3.7
**4**		+HCOO-	937.607	3.3	4.0	0.3	5.5	0.1
**5**	DGDG 32:1	+Cl-	925.566	1.5	0.3	3.6	3.7	11.4
**6**	DGDG 34:1	+Cl-	953.598	3.51	-0.4	19.5	16.8	0.2
**6**		+HCOO-	963.624	3.19	2.3	2.1	2.8	0.0
**6**		-H+	917.620	3.19	0.7	1.5	2.1	0.0
**7**	DGDG 34:2	+Cl-	951.581	3.14	0.4	4.1	9.0	1.2
**7**		-H+	915.605	4.36	-0.1	0.4	2.3	1.4
**8**	DGDG 34:3	+Cl-	949.566	1.5	0.0	0.7	1.3	0.0
**9**	DGDG 35:1	+Cl-	967.611	3.17	2.3	5.0	8.6	0.0
**10**	DGDG 36:0	+Cl-	983.642	1.55	2.3	1.4	0.5	0.8
**10**		+HCOO-	993.664	2.81	5.0	0.1	0.1	2.0
**11**	DGDG 36:1	+Cl-	981.627	3.08	1.3	18.1	7.4	1.6
**11**		+HCOO-	991.653	3.34	4.4	2.0	1.6	2.6
**12**	DGDG 36:2	+Cl-	979.613	1.48	0.3	7.3	5.5	0.0
**12**		+HCOO-	989.641	3.45	1.3	0.5	0.9	0.0
**12**		-H+	943.636	4.21	0.4	0.3	0.7	0.3
**13**	DGDG 36:3	+Cl-	977.599	1.48	-1.2	0.4	0.5	0.2
**14**	DGDG 36:4	-H+	939.595	3.24	5.0	0.5	1.4	1.7
**14**		+Cl-	975.583	1.31	-1.0	0.2	1.0	0.2

**15**	DGDG 37:5	-H+	951.611	3.24	-5.0	4.1	7.7	0.7
**16**	DGDG 38:0	+HCOO-	1021.714	4.83	5.0	0.0	0.0	19.4
**17**	DGDG 38:1	+Cl-	1009.657	2.99	2.6	3.0	1.3	31.8
**18**	DGDG 38:2	+Cl-	1007.645	3.36	-0.2	11.3	5.6	4.5
**18**		+HCOO-	1017.672	3.36	1.2	0.5	0.4	0.2
**18**		-H+	971.666	2.76	2.0	0.1	0.6	1.1
**19**	DGDG 38:3	+Cl-	1005.631	3.38	-2.0	1.2	0.8	0.0
**20**	DGDG 39:2	+Cl-	1021.659	3.04	1.2	0.5	0.5	2.6
**21**	DGDG 40:1	+Cl-	1037.679	2.95	5.0	0.5	0.1	0.6
**22**	DGDG 40:2	+Cl-	1035.673	1.48	2.1	3.4	1.6	2.1
**1**	NAPE 44:1	-H+	870.656	7.21	3.8	0.2	0.1	0.7
**2**	NAPE 44:2	-H+	868.642	7.19	1.6	0.1	0.1	1.2
**3**	NAPE 44:3	-H+	866.627	7.19	1.8	0.5	0.0	0.4
**4**	NAPE 46:1	-H+	898.689	7.21	1.8	0.3	0.5	0.9
**5**	NAPE 46:2	-H+	896.674	7.15	1.5	0.2	0.0	1.8
**6**	NAPE 46:3	-H+	894.658	7.13	1.5	0.1	0.0	1.0
**7**	NAPE 46:4	-H+	892.640	7.1	4.3	0.6	2.5	0.3
**8**	NAPE 48:1	-H+	926.721	7.32	0.7	1.1	0.0	1.5
**9**	NAPE 48:2	-H+	924.706	7.25	0.7	0.8	0.0	2.2
**10**	NAPE 48:3	-H+	922.690	7.12	0.9	0.1	0.0	1.0
**11**	NAPE 50:1	-H+	954.752	7.33	1.3	5.4	5.7	5.9
**12**	NAPE 50:2	-H+	952.737	7.25	0.9	4.7	2.1	4.8
**13**	NAPE 50:3	-H+	950.721	7.2	1.1	1.4	0.2	2.0
**14**	NAPE 50:4	-H+	948.705	7.04	1.7	0.2	0.5	0.6
**15**	NAPE 51:1	-H+	968.766	7.23	3.3	0.6	0.0	0.5
**16**	NAPE 51:2	-H+	966.751	7.15	2.2	0.7	0.0	0.8
**17**	NAPE 52:1	-H+	982.782	6.94	2.6	2.2	6.9	3.4
**18**	NAPE 52:2	-H+	980.768	7.25	1.2	17.8	16.9	21.6
**19**	NAPE 52:3	-H+	978.756	6.89	-2.5	7.7	6.1	13.2
**20**	NAPE 53:2	-H+	994.782	7.25	2.5	1.7	0.3	0.8
**21**	NAPE 53:3	-H+	992.766	7.08	2.8	0.4	0.0	0.6
**22**	NAPE 54:1	-H+	1010.807	7.29	5.0	3.9	1.7	1.9
**23**	NAPE 54:2	-H+	1008.800	6.89	-0.1	26.5	30.9	13.1
**24**	NAPE 54:3	-H+	1006.784	6.92	0.7	14.6	15.1	16.0
**25**	NAPE 54:4	-H+	1004.767	7.22	1.6	5.6	5.7	2.7
**26**	NAPE 54:5	-H+	1002.752	7.19	1.4	2.1	4.7	0.6
**27**	NAPE 56:3	-H+	1034.809	7.04	5.0	0.4	0.0	0.4
**1**	PG 26:0	-H+	637.408	7.79	1.0	0.6	0.1	0.7
**2**	PG 30:0	-H+	693.471	7.68	-0.1	2.4	0.6	2.3
**3**	PG 32:0	-H+	721.503	8.27	-0.6	0.9	7.7	4.3
**4**	PG 32:1	-H+	719.487	8.25	-0.6	0.0	5.0	0.3
**5**	PG 34:0	-H+	749.520	7.37	5.0	1.9	0.5	3.4
**6**	PG 34:1	-H+	747.519	7.53	-1.2	17.8	26.8	20.7
**7**	PG 34:2	-H+	745.504	8.2	-2.6	6.2	15.2	5.8
**8**	PG 34:3	-H+	743.488	8.2	-2.1	0.3	11.1	1.1
**9**	PG 35:1	-H+	761.534	8.22	0.1	10.4	3.6	4.3
**10**	PG 35:2	-H+	759.517	7.6	0.9	3.1	1.4	5.6
**11**	PG 36:0	-H+	777.550	7.33	5.0	0.6	0.3	2.2
**12**	PG 36:1	-H+	775.550	7.49	-0.1	18.1	12.0	18.5
**13**	PG 36:2	-H+	773.534	7.47	0.3	10.4	1.4	18.9
**14**	PG 36:3	-H+	771.518	7.45	0.3	3.4	0.5	6.2
**15**	PG 36:4	-H+	769.502	7.41	0.5	0.9	0.4	1.7
**16**	PG 36:5	-H+	767.488	8.16	-0.9	0.0	1.7	0.3
**17**	PG 38:1	-H+	803.581	7.57	-0.3	4.9	0.2	0.2
**18**	PG 38:2	-H+	801.566	8.15	-0.6	12.0	7.4	0.8
**19**	PG 38:3	-H+	799.549	7.55	0.8	1.6	1.6	1.7
**20**	PG 38:4	-H+	797.534	7.27	0.3	0.1	0.1	0.7
**21**	PG 40:2	-H+	829.597	8.12	-0.6	4.1	2.5	0.3
**1**	PI 30:0	-H+	781.486	8.45	1.1	0.6	0.4	0.4
**2**	PI 32:0	-H+	809.518	8.47	0.3	1.7	1.7	1.4
**3**	PI 32:1	-H+	807.502	8.39	0.8	1.3	1.3	1.0
**4**	PI 33:0	-H+	823.534	8.47	0.1	0.4	0.4	0.4
**5**	PI 33:1	-H+	821.518	8.39	0.7	0.5	0.4	0.4
**6**	PI 34:0	-H+	837.540	8.2	5.0	1.2	1.5	1.2
**7**	PI 34:1	-H+	835.534	8.37	-0.2	8.4	10.4	6.3
**8**	PI 34:2	-H+	833.518	8.33	0.1	2.1	3.6	1.8
**9**	PI 35:1	-H+	849.549	8.37	1.4	0.8	0.8	0.6
**10**	PI 35:2	-H+	847.533	8.32	1.2	0.4	0.4	0.3
**11**	PI 36:0	-H+	865.565	8.74	5.0	0.5	4.0	0.4
**12**	PI 36:1	-H+	863.565	8.37	1.1	32.2	30.7	30.5
**13**	PI 36:2	-H+	861.550	8.3	-0.6	25.8	23.2	24.7
**14**	PI 36:3	-H+	859.534	8.27	-0.2	4.3	4.8	3.9

**15**	PI 36:4	-H+	857.518	8.17	0.2	0.7	1.0	0.7
**16**	PI 37:2	-H+	875.558	8.72	5.0	0.6	0.5	0.4
**17**	PI 38:1	-H+	891.595	8.36	1.9	0.6	0.6	0.5
**18**	PI 38:2	-H+	889.580	8.26	1.6	1.4	1.2	1.2
**19**	PI 38:3	-H+	887.564	8.21	1.9	4.4	3.5	6.3
**20**	PI 38:4	-H+	885.549	8.13	1.2	6.7	5.3	12.1
**21**	PI 38:5	-H+	883.534	8.1	0.5	4.2	3.4	4.9
**22**	PI 38:6	-H+	881.517	8.12	1.4	0.7	0.6	0.3
**23**	PI 40:5	-H+	911.564	8.12	1.9	0.3	0.3	0.3
**1**	PE 28:0	-H+	634.446	10.01	-1.8	1.4	1.2	1.4
**2**	PE 30:0	-H+	662.478	9.95	-2.2	2.5	2.3	2.5
**3**	PE 30:1	-H+	660.464	9.92	-4.4	1.6	1.3	1.4
**4**	PE 31:0	-H+	676.493	9.93	-0.4	0.5	0.5	0.5
**5**	PE 32:0	-H+	690.509	9.84	-1.0	1.4	1.4	1.8
**6**	PE 32:1	-H+	688.493	9.87	-1.7	5.7	4.7	4.3
**7**	PE 32:2	-H+	686.482	9.85	-5.0	1.6	1.5	1.5
**8**	PE 33:1	-H+	702.509	9.85	-1.0	0.9	0.7	0.6
**9**	PE 34:1	-H+	716.524	9.82	-0.6	14.0	13.7	11.5
**10**	PE 34:2	-H+	714.509	9.81	-2.0	9.2	9.2	9.0
**11**	PE 34:3	-H+	712.496	9.81	-4.8	1.4	3.3	0.9
**12**	PE 35:1	-H+	730.539	9.8	0.4	0.6	0.7	0.5
**13**	PE 36:1	-H+	744.554	9.74	1.5	4.8	5.9	5.0
**14**	PE 36:2	-H+	742.539	9.69	0.1	22.7	22.2	22.2
**15**	PE 36:3	-H+	740.525	9.69	-1.7	9.0	9.5	11.0
**16**	PE 36:4	-H+	738.510	9.63	-2.3	2.9	3.6	3.1
**17**	PE 38:3	-H+	768.552	9.59	3.9	0.7	0.7	1.0
**18**	PE 38:4	-H+	766.539	9.54	-0.3	3.8	3.7	5.3
**19**	PE 38:5	-H+	764.524	9.53	-1.2	5.1	4.7	5.3
**20**	PE 38:6	-H+	762.510	9.53	-3.2	1.5	1.4	0.9
**21**	PE 40:5	-H+	792.555	9.53	-0.4	1.2	1.1	1.3
**22**	PE 40:6	-H+	790.539	9.52	0.0	1.8	1.7	1.2
**1**	PEO 34:2	-H+	700.520	9.65	5.0	0.9	0.7	0.8
**2**	PEO 34:3	-H+	698.513	9.63	-0.5	0.6	0.5	0.9
**3**	PEO 36:3	-H+	726.544	9.58	0.6	0.8	0.7	0.9
**4**	PEO 36:4	-H+	724.529	9.56	-0.4	0.6	0.6	0.9
**5**	PEO 36:5	-H+	722.514	9.48	-1.9	1.0	0.9	1.7
**6**	PEO 36:6	-H+	720.498	9.53	-1.2	0.5	0.4	0.3
**7**	PEO 38:5	-H+	750.545	9.41	-0.6	0.4	0.4	1.1
**8**	PEO 38:6	-H+	748.529	9.41	-0.3	0.8	0.7	1.1
**1**	LPE 16:0	-H+	452.279	12.29	-2.0	2.9	3.9	9.8
**2**	LPE 16:1	-H+	450.264	12.38	-3.8	2.7	1.7	1.1
**3**	LPE 17:1	-H+	464.285	12.15	-5.0	0.6	0.2	0.3
**4**	LPE 18:0	-H+	480.310	11.95	-1.4	7.0	14.5	13.9
**5**	LPE 18:1	-H+	478.294	12.02	-0.2	39.5	34.7	53.8
**6**	LPE 18:2	-H+	476.279	12.15	-1.1	14.4	13.0	14.3
**7**	LPE 18:3	-H+	474.263	12.27	-1.3	0.8	2.4	0.6
**8**	LPE 20:0	-H+	508.343	12.54	-3.5	0.8	5.3	0.0
**9**	LPE 20:3	-H+	502.294	11.96	-0.7	3.8	2.6	1.3
**10**	LPE 20:4	-H+	500.279	11.91	-1.3	5.8	3.8	1.5
**11**	LPE 20:5	-H+	498.263	12.29	-0.1	1.4	1.1	0.2
**12**	LPE 21:3	-H+	516.303	13.22	5.0	0.6	4.0	0.0
**13**	LPE 22:0	-H+	536.374	12.37	-3.4	0.1	2.8	0.0
**14**	LPE 22:5	-H+	526.295	12.17	-1.4	3.8	2.7	0.7
**15**	LPE 24:0	-H+	564.408	12.2	-4.4	0.0	2.0	0.0
**1**	LPEO 13:1	-H+	394.237	13.55	-1.6	0.6	0.5	0.0
**2**	LPEO 14:1	-H+	408.253	13.33	-1.2	1.1	0.6	0.1
**3**	LPEO 15:1	-H+	422.267	13.18	2.3	4.2	1.1	0.4
**4**	LPEO 16:0	-H+	438.299	13.3	-0.1	1.1	0.5	0.1
**5**	LPEO 16:1	-H+	436.284	11.81	-0.8	6.5	1.2	1.3
**6**	LPEO 18:1	-H+	464.314	11.54	1.3	2.3	1.4	0.7
**1**	PS 32:0	-H+	734.489	9.75	5.0	0.3	0.7	0.2
**2**	PS 34:1	-H+	760.514	10.78	-1.4	5.6	5.2	3.2
**3**	PS 34:2	-H+	758.498	10.72	-0.9	1.7	1.5	1.7
**4**	PS 35:2	-H+	772.517	9.57	-4.6	0.6	0.6	0.3
**5**	PS 36:0	-H+	790.547	10.82	5.0	4.0	4.2	3.3
**6**	PS 36:1	-H+	788.545	10.75	-1.0	35.3	38.1	33.6
**7**	PS 36:2	-H+	786.531	10.65	-2.4	31.3	30.0	31.9
**8**	PS 36:3	-H+	784.516	10.61	-3.2	6.3	6.1	6.7
**9**	PS 36:4	-H+	782.499	10.57	-1.8	0.7	0.7	0.6
**10**	PS 38:3	-H+	812.545	10.56	-0.7	2.5	3.0	3.7
**11**	PS 38:4	-H+	810.528	10.61	0.9	2.5	2.0	3.3
**12**	PS 38:5	-H+	808.512	10.61	1.9	2.2	1.5	3.7

**13**	PS 38:6	-H+	806.495	10.58	3.4	0.5	0.3	0.8
**14**	PS 40:4	-H+	838.559	10.47	2.1	0.6	0.6	1.4
**15**	PS 40:5	-H+	836.545	10.44	0.0	4.4	4.1	4.1
**16**	PS 40:6	-H+	834.530	10.42	-0.7	1.6	1.4	1.4
**1**	PC 26:0	-CH3+	634.446	15.65	-1.3	2.4	1.7	2.5
**2**	PC 26:1	-CH3+	632.430	15.66	-1.0	0.2	0.1	0.2
**3**	PC 27:0	-CH3+	648.462	15.59	-1.2	0.3	0.2	0.3
**4**	PC 28:0	-CH3+	662.477	15.54	-1.2	10.8	9.1	11.6
**5**	PC 29:0	-CH3+	676.492	15.51	0.2	2.0	1.7	2.0
**6**	PC 30:0	-CH3+	690.508	15.47	-0.1	27.1	26.4	26.5
**7**	PC 30:1	-CH3+	688.494	15.48	-3.0	1.3	1.6	1.1
**8**	PC 31:0	-CH3+	704.523	15.44	0.5	3.2	3.2	2.9
**9**	PC 31:1	-CH3+	702.508	15.44	0.4	0.3	0.3	0.3
**10**	PC 32:0	-CH3+	718.539	15.39	-0.3	10.9	10.8	11.2
**11**	PC 32:1	-CH3+	716.525	15.36	-1.5	5.4	5.5	4.7
**12**	PC 32:2	-CH3+	714.510	15.38	-3.4	0.7	1.1	0.8
**13**	PC 33:0	-CH3+	732.555	15.36	0.1	0.6	0.6	0.6
**14**	PC 33:1	-CH3+	730.539	15.35	0.4	1.0	0.9	0.8
**15**	PC 34:0	-CH3+	746.561	15.29	5.0	1.3	1.3	1.3
**16**	PC 34:1	-CH3+	744.556	15.28	-1.0	11.8	10.5	9.5
**17**	PC 34:2	-CH3+	742.541	15.29	-1.9	4.2	5.0	4.9
**18**	PC 34:3	-CH3+	740.523	15.34	0.4	0.8	2.4	0.4
**19**	PC 35:1	-CH3+	758.569	15.25	1.7	0.4	0.3	0.3
**20**	PC 35:2	-CH3+	756.555	15.24	-0.4	0.3	0.3	0.2
**21**	PC 36:1	-CH3+	772.585	15.19	1.2	1.8	1.8	1.7
**22**	PC 36:2	-CH3+	770.571	15.17	-0.9	5.8	6.7	6.7
**23**	PC 36:3	-CH3+	768.556	15.19	-1.2	2.5	2.9	3.3
**24**	PC 36:4	-CH3+	766.540	15.13	-0.6	1.3	2.4	1.7
**25**	PC 38:2	-CH3+	798.596	15.02	5.0	0.4	0.4	0.2
**26**	PC 38:3	-CH3+	796.583	15.05	4.5	0.3	0.3	0.4
**27**	PC 38:4	-CH3+	794.571	15.04	-1.1	0.6	0.6	1.1
**28**	PC 38:5	-CH3+	792.569	14.92	-5.0	1.7	1.5	2.2
**29**	PC 38:6	-CH3+	790.538	15.01	1.2	0.3	0.3	0.4
**30**	PC 39:4	-CH3+	808.600	15.31	-5.0	0.3	0.2	0.2
**1**	LPC 10:0	-CH3+	396.218	18.17	-4.9	0.3	0.3	0.0
**2**	LPC 12:0	-CH3+	424.248	17.77	-3.4	0.2	0.0	0.1
**3**	LPC 14:0	-CH3+	452.279	17.45	-2.4	1.9	1.0	3.2
**4**	LPC 15:0	-CH3+	466.296	17.33	-4.9	1.0	0.2	1.3
**5**	LPC 16:0	-CH3+	480.310	17.25	-1.4	37.6	25.0	38.2
**6**	LPC 17:0	-CH3+	494.324	17.15	1.6	2.4	0.7	1.5
**7**	LPC 17:1	-CH3+	492.315	17.21	-5.0	2.7	0.6	0.5
**8**	LPC 18:0	-CH3+	508.342	17.03	-1.9	24.5	30.9	13.1
**9**	LPC 18:1	-CH3+	506.326	17.05	-1.4	14.2	21.8	29.5
**10**	LPC 18:2	-CH3+	504.310	17.16	-1.4	5.1	8.4	9.5
**11**	LPC 18:3	-CH3+	502.294	17.24	-0.6	0.5	3.3	0.6
**12**	LPC 19:0	-CH3+	522.357	16.89	0.0	0.5	0.4	0.1
**13**	LPC 19:1	-CH3+	520.341	16.98	0.5	0.2	0.1	0.2
**14**	LPC 20:0	-CH3+	536.374	16.81	-3.1	5.8	5.7	0.2
**15**	LPC 20:1	-CH3+	534.358	16.91	-2.7	1.0	0.9	0.2
**16**	LPC 20:2	-CH3+	532.338	16.97	5.0	0.2	0.2	0.2
**17**	LPC 20:3	-CH3+	530.323	17.01	3.3	1.1	0.2	0.8
**18**	LPC 20:4	-CH3+	528.309	17.01	0.4	1.0	0.1	0.7
**1**	SM 30:0;2	-CH3+	633.497	16.49	0.9	0.6	0.5	0.6
**2**	SM 30:1;2	-CH3+	631.484	16.62	-2.4	1.0	0.9	1.1
**3**	SM 31:0;2	-CH3+	647.507	16.46	5.0	0.1	0.1	0.1
**4**	SM 31:1;2	-CH3+	645.499	16.56	-2.0	0.6	0.6	0.6
**5**	SM 32:0;2	-CH3+	661.527	16.39	2.5	4.9	4.9	4.5
**6**	SM 32:1;2	-CH3+	659.514	16.51	-1.6	17.6	16.8	18.8
**7**	SM 32:2;2	-CH3+	657.499	16.52	-2.7	0.1	0.1	0.1
**8**	SM 33:0;2	-CH3+	675.540	16.37	5.0	1.2	1.3	1.1
**9**	SM 33:1;2	-CH3+	673.529	16.46	-0.4	6.9	6.9	6.4
**10**	SM 33:2;2	-CH3+	671.513	16.42	0.5	0.2	0.2	0.1
**11**	SM 34:0;2	-CH3+	689.557	16.31	4.9	7.4	7.6	7.4
**12**	SM 34:1;2	-CH3+	687.545	16.4	0.0	35.9	36.0	35.0
**13**	SM 34:2;2	-CH3+	685.529	16.41	-0.7	1.3	1.2	1.2
**14**	SM 35:1;2	-CH3+	701.560	16.35	0.7	1.8	1.8	1.7
**15**	SM 36:0;2	-CH3+	717.585	16.25	5.0	0.4	0.4	0.6
**16**	SM 36:1;2	-CH3+	715.577	16.29	-0.9	2.5	2.5	4.1
**17**	SM 36:2;2	-CH3+	713.561	16.31	-0.4	0.4	0.4	0.6
**18**	SM 37:1;2	-CH3+	729.592	16.25	-0.4	0.5	0.5	0.6
**19**	SM 38:1;2	-CH3+	743.609	16.19	-2.2	3.9	4.0	4.0
**20**	SM 38:2;2	-CH3+	741.593	16.21	-1.6	0.1	0.1	0.1

**21**	SM 39:0;2	-CH3+	759.635	16.04	4.3	0.9	0.9	0.8
**22**	SM 39:1;2	-CH3+	757.623	16.16	0.1	3.2	3.2	2.5
**23**	SM 39:2;2	-CH3+	755.600	16.19	5.0	0.5	0.4	0.2
**24**	SM 40:1;2	-CH3+	771.639	16.12	-0.3	3.2	3.5	3.2
**25**	SM 40:2;2	-CH3+	769.623	16.14	0.5	0.6	0.6	0.4
**26**	SM 41:0;2	-CH3+	787.665	16.01	5.0	0.4	0.4	0.4
**27**	SM 41:1;2	-CH3+	785.654	16.08	0.1	1.7	1.9	1.8
**28**	SM 41:2;2	-CH3+	783.638	16.09	0.3	0.5	0.4	0.2
**29**	SM 42:1;2	-CH3+	799.670	16.03	-0.5	1.2	1.2	1.2
**30**	SM 42:2;2	-CH3+	797.654	16.05	0.2	0.4	0.4	0.3
**31**	SM 43:1;2	-CH3+	813.684	15.98	1.4	0.2	0.2	0.2

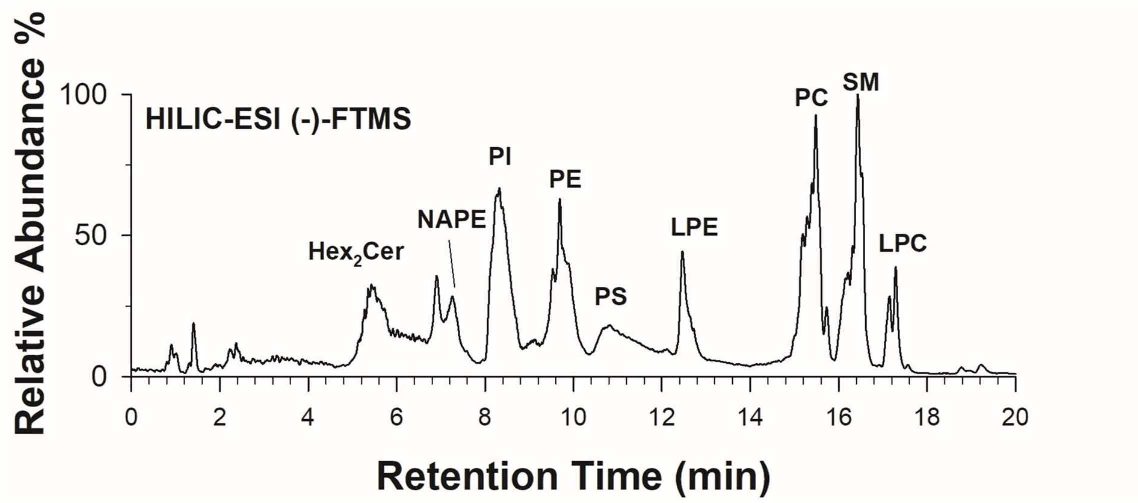

Figure S1. Representative total ion current (TIC) chromatogram in negative ion mode of a lipid extract from bovine milk.

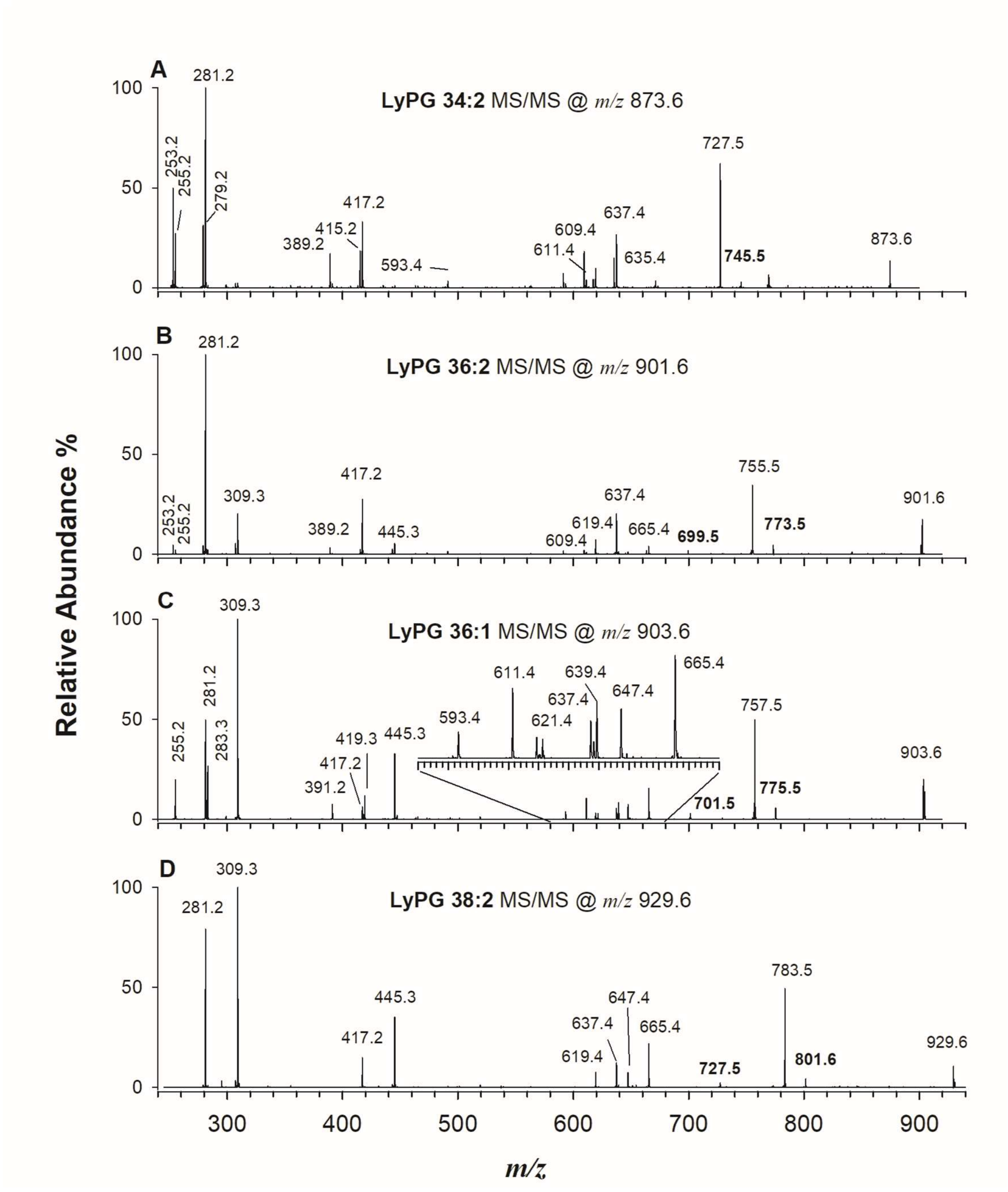

Figure S2. Some examples of MS/MS spectra collected for the major LyPG retrieved in kefir sample at *m/z* 873.6 (A), 901.6 (B), 903.6 (C), 929.6 (D).

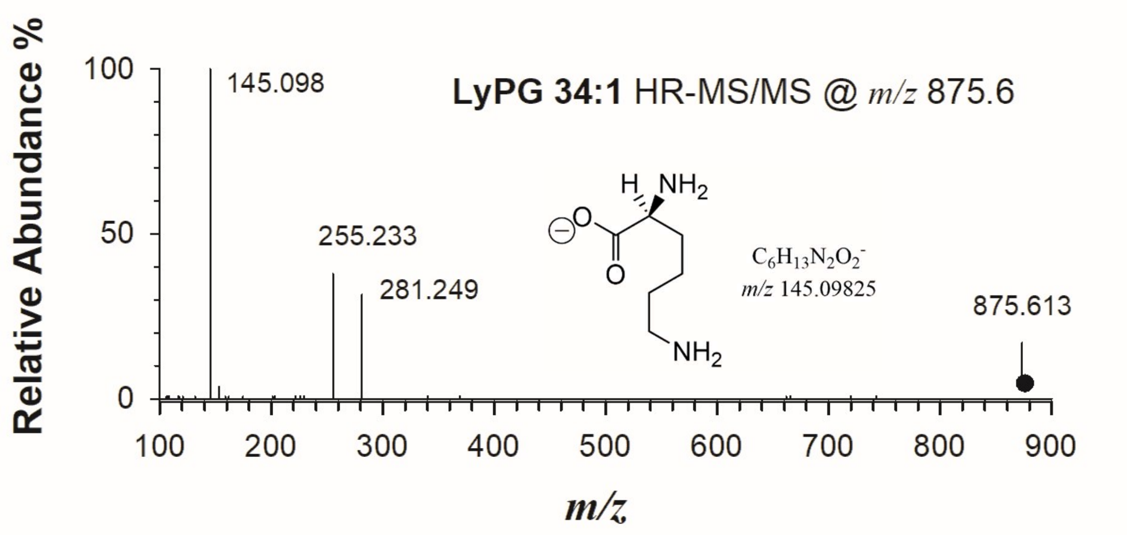

Figure S3: High-resolution MS/MS spectrum of LyPG 34:1. The inset shows the structure of the diagnostic ion at *m/z* 145.098.
